# Stocking impacts the expression of candidate genes and physiological condition in introgressed brook charr (*Salvelinus fontinalis*) populations

**DOI:** 10.1111/eva.12022

**Published:** 2012-10-23

**Authors:** Fabien C Lamaze, Dany Garant, Louis Bernatchez

**Affiliations:** 1 Département de Biologie, Institut de Biologie Intégrative et des Systèmes (IBIS), Université LavalQuébec, QC, Canada; 2 Département de Biologie Faculté des Sciences, Pavillon des Sciences de la vie, Université de SherbrookeSherbrooke, QC, Canada

**Keywords:** brook charr, gene expression, hybridization, introgression, quantitative PCR, stocking

## Abstract

Translocation of plants and animal populations between environments is one of the major forms of anthropogenic perturbation experienced by pristine populations, and consequently, human-mediated hybridization by stocking practices between wild and exogenous conspecifics is of increasing concern. In this study, we compared the expression of seven candidate genes involved in multifactorial traits and regulatory pathways for growth as a function of level of introgressive hybridization between wild and domestic brook charr to test the null hypothesis of no effect of introgression on wild fish. Our analyses revealed that the expression of two of the genes tested, cytochrome c oxidase VIIa and the growth hormone receptor isoform I, was positively correlated with the level of introgression. We also observed a positive relationship between the extent of introgression and physiological status quantified by the Fulton's condition index. The expression of other genes was influenced by other variables, including year of sampling (reflecting different thermal conditions), sampling method and lake of origin. This is the first demonstration in nature that introgression from stocked populations has an impact on the expression of genes playing a role in important biological functions that may be related with fitness in wild introgressed populations.

## Introduction

Translocation of plants and animal populations between environments is one of the major forms of anthropogenic perturbation experienced by pristine populations (Suarez and Tsutsui 2008; Laikre et al. 2010), which often result in hybridization between native and exogenous individuals (Allendorf et al. 2001; Randi 2008). Effects of hybridization can be harmful (Rhymer and Simberloff 1996; Allendorf et al. 2001; Laikre et al. 2010), and as a consequence, human-mediated hybridization by stocking practices, between individuals from wild populations and their exogenous conspecifics, is of increasing concern (Allendorf et al. 2001; Edmands 2007; Randi 2008). These concerns are exacerbated when exogenous conspecifics are propagated in captivity through breeding programmes.

Domestication has been practiced for millennia on numerous species for traits advantageous to humans (Diamond 2002; Mignon-Grasteau et al. 2005; Taberlet et al. 2008). The main consequences of domestication are the rapid genetic and phenotypic changes that wild species undergo through the process of artificial selection (Jensen 2006). The process of domestication can also be perceived as fast human-induced evolution leading to rapid changes in the genetic architecture, which is the sum of genetic interactions responsible for the development of characters in strains under selection (Burger et al. 2008).

Since the 1970s, directed selective breeding programmes towards domestication have been undertaken in several salmonid species, such as Atlantic salmon (*Salmo salar*) and rainbow trout (*Oncorhynchus mykiss*; Gjedrem 2000). These breeding programmes have improved traits of commercial interest, such as faster growth and improved disease resistance (Gjoen and Bentsen 1997; Gjedrem 2000). Other non-target genetically based phenotypes have also been modified, including physiological, morphological and behavioural changes, such as increased fat content and aggressiveness (Fleming and Einum 1997; Fleming et al. 2002; Tymchuk et al. 2007). The latter changes typically resulted in poorer performance of domesticated individuals in the wild (McGinnity et al. 2003; Araki et al. 2009).

Modulation of genes expression is increasingly recognized as playing an important role in phenotypic changes (Oleksiak et al. 2002; Fay and Wittkopp 2008; Gibson 2008; Pavey et al. 2010). For example, parallelism in patterns of gene expression resulting from artificial selection has been documented in both European and North American Atlantic salmon strains (Roberge et al. 2006). Moreover, only three to seven generations of artificial selection can lead to heritable changes in gene transcription between domesticated versus wild populations (Roberge et al. 2006; Sauvage et al. 2010). Biological functions affected by artificial selection include immunity, transport, fatty acids and steroids metabolism, carbohydrate metabolism, protein and nucleic acid metabolism and cellular cycle/growth (Devlin et al. 2009; Normandeau et al. 2009; Bougas et al. 2010; Sauvage et al. 2010). Other studies revealed that genetic introgression from domestic to wild populations caused gene misregulation as a consequence of rapid evolutionary changes accumulated during domestication (Roberge et al. 2008; Normandeau et al. 2009). Moreover the extent and patterns of gene misregulation observed in first hybrid generations has been shown to vary with the specific genomic architecture of the wild and domestic strains (Bougas et al. 2010).

Brook charr (*Salvelinus fontinalis*) is one of the most economically important species for freshwater aquaculture in North Eastern America, for both food and stocking purposes to support recreational fisheries (Page and Burr 1991). For example, in the province of Québec (Canada) only, six millions of hatchery (or domestic) brook charr are stocked every year to support the angling industry (Ministère des Ressources Naturelles et de la Faune du Québec 2008). Recent studies showed that stocking intensity have impacted the genetic integrity of wild populations by altering intrapopulation genetic diversity and homogenizing population genetic structure (Marie et al. 2010; Lamaze et al. 2012). By means of coding-gene SNP genotyping, Lamaze et al. (2012) showed that the dynamic of domestic introgression into wild populations was mediated by either positive or negative selection depending on loci. This study further suggested that genes involved in growth-related biological functions were over-represented among loci for which introgression was apparently under selection.

This study expands upon previous research efforts on brook charr using a quantitative PCR (qPCR) approach to compare patterns of gene transcription in the wild (Marie et al. 2010; Lamaze et al. 2012). More specifically, we compared expression of seven candidate genes involved in multifactorial traits and regulatory pathways for growth as a function of level of introgressive hybridization between wild and domestic fish. We tested the null hypothesis of no effect of domestic introgression on patterns of gene expression in wild fish. Numerous studies have documented patterns of introgression due to stocking at the DNA level either based on neutral or to a lesser extent on potentially selected loci (Hansen et al. 2010; Marie et al. 2010; Bourret et al. 2011; Lamaze et al. 2012). To our knowledge, this is the first study that documents the effect of stocking on patterns of gene expression in natural conditions and provides empirical demonstration of functional impacts of stocking in a conservation genetics context.

## Materials and Methods

### Sampling strategy

Two lakes were selected in two provincial reserves, on the basis of their known levels of recent fish stocking and the *a priori* knowledge about the presence of both hybrid and pure fish within the same lake (Marie et al. 2010). In the Mastigouche wildlife reserve, hybrids (F1) between female of the domestic strain [Lac des Écorces aquaculture facility (LDE), Lac-Des-Écorces, QC] and a wild strain male [Bourassa Lake (BOU), Mastigouche reserve] were used for stocking, whereas only a second pure domestic strain [Jacques Cartier aquaculture facility (JC), Cap-Santé, QC] was used in the Portneuf Wildlife Reserve. Live brook charr were caught in May and June using three methods: trap nets, gillnets and angling. Experimental gillnets with different mesh size were used (1.8 m height × 38 m length). Traps were large enough (5 m length × 1.8 m width × 1.4 m height) and included size-selecting compartments to reduce confinement and predation. Gillnets and traps were checked at the maximum every 40 min and 8 h, respectively. In the Petit Saint-Bernard Lake (BER; Mastigouche reserve, QC, Canada, 46°33′50″N, 73°18′54″W), 14 and 47 brook charr were captured in 2008 and 2009, respectively. In the Méthot Lake (MET; Portneuf reserve, QC, Canada, 47°10′21″N, 72°19′32″W), 27 brook charr in 2008 and 43 in 2009 were sampled. The sex ratio (F/M) for fish that could unambiguously be classified as female and male was 37/23 and 28/15 for BER and MET, respectively. All fish were acclimatized for 20 min in a dark tank prior to euthanasia with eugenol (purity: 99%) (Sigma Inc., St. Louis, MO, USA) for 15 min at a final concentration of 75 mg/L. Liver tissue was sampled from each individual within a minute following death and was preserved in RNA later® (2008; Qiagen, Mississauga, ON, Canada) or frozen in liquid nitrogen (2009) and later stored at −80°C for subsequent RNA analysis. In addition, adipose fins were clipped from each individual and stored in 95% ethanol for DNA analysis. Additionally, fork length (mm), weight (g) and sex were recorded. Adipose fins from reference-stocked domestic strains were sampled from 40 individuals of LDE and BOU, and 51 individuals from JC in 2007.

### DNA and RNA extraction

Total DNA was extracted from 20 mg of adipose fins tissue using a salt-extraction protocol (Aljanabi and Martinez 1997). DNA quality was evaluated by electrophoresis on a 1% agarose gel, and DNA concentrations as well as purity were determined by spectrophotometry using a Multiskan® Spectrum (Thermo labsystems, Waltham, MA, USA). Total RNA was extracted from 20 mg of the liver tissue homogenized using a tissue lyser (Qiagen) at 30 Hz for 5 min, using the PureLink™ RNA mini kit protocol (Invitrogen, Burlington, VT, USA) with DNAse I treatment during RNA purification, applied directly on the membrane of the column according to the manufacturer's protocol. Then samples were RNase treated as described by the manufacturer and stored at −80°C.

### cDNA synthesis

RNA was quantified using a NanoDrop 2000 spectrophotometer (Thermo Fisher Scientific Inc., Wilmington, DE, USA), and its quality was assessed using the Experion RNA HighSens Analysis Kit on an Experion automated electrophoresis station (BioRad, Mississauga, ON, Canada). First-strand cDNA was then synthesized from 5 μg of total RNA using the high-capacity cDNA Reverse Transcription Kit (Applied Biosystems, Foster City, CA, USA), according to the manufacturer's instructions. The ribosomal 18S gene (Human Euk 18S rRNA; Applied Biosystems) was used as the endogenous reference gene.

### PCR amplification of genomic DNA

To estimate the introgression level for each individual, a total of 23 microsatellites marker were genotyped for all sampled individuals [*Sco*216, *Sco*218 (Dehaan and Ardren 2005); *Sfo*177, *Sfo*226, *Sfo*262, *Sfo*266, *Sfo*269, *Sfo*308 (Perry et al. 2005); *Sfo*B52, *Sfo*C24, *Sfo*C28, *Sfo*C86, *Sfo*C88, *Sfo*C113, *Sfo*C115, *Sfo*D75, *Sfo*D91, *Sfo*D100, *Sfo*D105 (T. L. King, unpublished data); *Ssa*85, *Ssa*197 (O'Reilly et al. 1996); *One*8 (Scribner et al. 1996); *Sfo*12 (Angers et al. 1995)]. All these markers except *One*8 have previously been mapped (Sauvage et al. 2012a). Among them two markers were within the 95% confidence interval of QTLs. *Sfo*D75 is linked to growth rate, weight, transcription level of the insulin growth factor 1 receptor gene and hepato-somatic index. *Sfo*D105 is linked to hepato-somatic index. None of the markers used were linked, except for *Sfo*269 and *Sfo*C115 that were on the same chromosome and separated by a distance of 0.914 cM (Sauvage et al. 2012a,b). PCR amplification was performed using 2–10 ng of genomic DNA in a final reaction volume of 10 μL. PCR amplifications were multiplexed (Table S1) using Qiagen® Multiplex PCR kit (Qiagen Inc.) as recommended by the manufacturer, and primers were labelled using four different labels (FAM; Sigma-Aldrich® and VIC, NED and PET Applied Biosystems). The PCR protocol consisted of a 95°C activation step for 15 min, followed by 34 cycles of a denaturation step of 94°C for 30 s, an annealing step for 3 min where the temperature depended on the multiplexed primers (Table S1), and elongation step at 72°C for 1 min. A final extension step at 72°C for 10 min and a cool-down step at 10°C were added. A positive and a negative control were included in each PCR set of PCR experiment. PCR products were run on an ABI 3100 Genetic Analyser (Applied Biosystems).

### qPCR genes selection, primer design and amplification

Seven genes previously identified in microarray experiments as putative candidates being involved in the differentiation between domestic and wild populations were analysed in this study (Roberge et al. 2008; Normandeau et al. 2009; Bougas et al. 2010; Sauvage et al. 2010): *S. salar* apolipoprotein A-IV precursor (BT047267.1); *S. salar* cytochrome c oxidase VIIa (COX VIIa; BT048206.1); *S. salar* heat shock protein 90 (BT043623.1); *S. salar* metallothionein B (BT047801.1); *O. mykiss* transferrin (NM_001124552.1). In addition, we included insulin-like growth factor I and the growth hormone receptor isoform I (GHR-I) which were originally described for qPCR in brook charr and developed from *Oncorhynchus keta* (AF063216.1) and *Oncorhynchus kisutch* (AF403539.1), respectively (Côté et al. 2007). *Oncorhynchus mykiss* beta actin (NM_001124235.1) and *O. mykiss* elongation factor I (NM_001124339.1) were included as reference genes, in addition to 18S gene. These were chosen for their stability from previously described gene expression experiments (Olsvik et al. 2005; Ingerslev et al. 2006; Jeukens et al. 2009; Pierron et al. 2009; Croisetière et al. 2010). Sequences were retrieved from GeneBank with similarity searches with blast. For each gene, non-specific primers were designed from the coding regions of salmonid species using the amplifx software, v1.5.4 (http://en.bio-soft.net/pcr/AmplifX.html; Table S2). The use of cDNA and DNA allowed identifying intronic regions to design-specific qPCR primers around splicing sites, ensuring higher accuracy of cDNA amplification. Thus, cDNA and DNA of five individuals from each of the two experimental populations were amplified by PCR using the GoTaq® Flexi DNA polymerase kit (Promega Corporation, Madison, WI, USA) in a final reaction volume of 12.5 μL. The PCR protocol for sequencing primers was a 95°C initial denaturation step for 2 min, followed by 35 cycles of a denaturation step of 94°C for 30 s, an annealing step for 30 s at 55°C, and elongation step at 72°C for 1 min. A final extension step at 72°C for 10 min and a cool-down step at 10°C were added. PCR products were screened on 2% agarose gel and specific amplicons were sequenced on both side on an ABI 3100 (Applied Biosystems) using the big Dye terminator v3 chemistry (Applied Biosystems). In samples that showed non-specific PCR products, each band of expected size was excised and gel-purified using the QIAquick Gel Extraction kit (Qiagen Inc.) prior to Sanger sequencing. Electropherogram from all sequences were checked for quality control prior to alignments using the bioedit software (Hall, Carlsbad, CA, USA). Similarity searches were performed on NCBI using blastn/blastx options to verify every obtained sequence (http://www.ncbi.nlm.nih.gov/).

For each generated sequence, specific primers and fluorescent Taqman minor groove binder (MGB) probes were designed from the sequence showing the highest *E*-value using the default parameters of the TaqMan® MGB quantification tool option within the primer express v3.0 software (Applied Biosystems). Sequences of specific primers and probes are reported in Table S2. Primers were tested using conventional PCR and tested by amplifying a single band of approximately 60–90 bp. The qPCRs were performed using a lightcycler®480 (F. Hoffmann-La Roche AG, Basel, Switzerland), and efficiencies for all pairs of primer and probe were calculated following the manufacturer's instructions. Efficiency values were sufficient to allow direct comparison of amplification plots according to the *ΔΔC*_*p*_ method or *E*-method. The qPCR amplifications were performed using the TaqMan® Gene expression Master Mix (Applied Biosystems) in a reaction volume of 15 μL using the standard amplification protocol of 50°C for 2 min, 95°C for 10 min, followed by 40 cycles of a denaturation step at 95°C for 15 s and an annealing/elongation step at 60°C for 1 min. An Eppendorf ® ep*Motion™* 5075 Workstation (Eppendorf, Hamburg, Germany) was used to load the 384-well plate. All samples were run in triplicate with non-template controls. Beta actin, elongation factor 1 and ribosomal 18S genes were evaluated as reference genes to normalize the results before calculating the relative expression levels. The stability of the reference genes was evaluated with the bestkeeper software tool (Pfaffl et al. 2004). As beta actin showed the lowest variability in our samples, this gene was finally retained as the most accurate endogenous reference.

### Genetic variation

All 23 microsatellites were manually scored using the genscan software (Applied Biosystems) and binned using the flexibin v2 software (Amos et al. 2007). A script was developed with the python v2.6.3 software (http://www.python.org/) to replace the output ordinal format from the flexibin to the true allele sizes. The frequency of null alleles or large alleles dropout was estimated with the micro-checker software (van Oosterhout et al. 2004). The subsequent analyses were conducted using fstat v2.9.3.2 (Goudet 1995). Intra-population genetic diversity and structure was quantified by expected (*H*_*E*_) and observed (*H*_*O*_) heterozygosity, allelic richness, *Ar* (based on the smallest sample size = 37), and excess or deficit in heterozygosites (*F*_IS_; Weir and Cockerham 1984). Population differentiation (*F*_ST_; Weir and Cockerham 1984) was calculated between pairs of samples with 1000 permutation for significance. Deviations from Hardy–Weinberg equilibrium (HWE) were assessed. HWE and pairwise *F*_ST_ tests were corrected for multiple testing by applying a Bonferroni correction.

### Bayesian clustering analyses

Individual genomic proportion (*Q*) was assessed using the Bayesian clustering software structure v2.3.1. (Pritchard et al. 2000). The estimated individual admixture was further used as independent variable in a multivariate statistical model. Individuals from the two experimental lakes as well as the three reference populations, including two domestic strains, were analysed together. The most likely number of clusters *K* in all simulations was assumed to be in the range of *K* = 1 to *K* = *n* + 3 (where *n* is the number of populations sampled; Evanno et al. 2005). Ten replicates were conducted for each *K* with a burn-in period of 1 × 10^4^, followed by 5 × 10^4^ MCMC steps. The *ad hoc* statistic Δ*K* was used to determine the most probable *K* (Evanno et al. 2005). An admixture model with correlated allele frequencies between populations was used with 1 × 10^5^ steps burnin-period followed by 5 × 10^5^ steps of the Markov Chain. All loci were included in the analysis including three that were not under HWE in some populations (see Results) as this had no effect on *Q* values estimates and downstream statistical analysis.

### Fulton's condition index

Fulton's condition index is commonly used to assess the general physiological condition in fishes (Ricker 1975). Higher values of this index are interpreted as reflecting greater energy reserves (Stevenson and Woods 2006): as a rule of thumb, a value <1 is often considered to reflect ‘skinny’ fish condition and values >1 as reflecting a more ‘fatty’ fish condition. The relationship between the mass and length was examined with a nonlinear regression to confirm isometric growth because length is raised to the third power in the equation. The nonlinear regression was 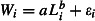 for all fish combined, where *W*_*i*_ is the mass (g) of the *i*th individual, *L*_*i*_ is its fork length (mm), and ε is a normally distributed error term. The Fulton's condition index was calculated as followed: *K* = *W*/*L*^3^ × 10^5^, where *W* represents the mass (g) and *L* the fork length (mm). A linear model using a generalized least squares (GLS) was applied, allowing for unequal variances of condition index among lakes. This model was used to test for any differences in the condition index, between lakes and years and along the admixture level of fish. The significance of fixed effects (α = 0.05) was obtained through a type III test (*F* statistic).

### Statistical analyses

All statistics were performed in r v2.14.1 (R Development Core Team, http://www.R-project.org). Normality of the gene expression data set was rejected by the Shapiro–Wilk test and thus a log_10_ transformation was applied on the dependant variables (gene expression level for each candidate gene). For both sampling years and lakes, a multivariate analysis of variance (manova) model was used to determine the statistical significance (α = 0.05; type III test) associated with the effect of six independent variables: (i) proportion of domestic admixture of each fish estimated with structure (*K* = 2), (ii) sex, (iii) Fulton's condition index, (iv) lake of origin, (v) year of sampling and (vi) sampling method (angling, gillnet, trap net), on the dependent variables. The manova model was the following:


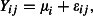


where *Y*_*ij*_ is the genes expression level for the *i*th individual and the *j*th gene, μ_*i*_ is the mean of all independent effects for the *i*th individual and ε_*ij*_ is the error term.

To visualize and confirm gene transcription significantly affected by the proportion of domestic admixture in the manova, a factorial analysis of mixed data (FAMD) was used with the package factominer (Lê et al. 2008). This method enables the incorporation of variables that may be continuous and/or categorical in a single analysis. Only significant independent variables from the manova model were included. The continuous variables were centred to a mean of zero and a variance of one before being used in the analysis. As the Fulton's condition index covaries with the year of sampling (see Results), it was normalized for each year prior performing the FAMD to remove the year effect.

## Results

### Genetic variation and differentiation

All 23 microsatellites were highly polymorphic, with an average of 17 (±16) alleles per locus with a range of 4–85 alleles. Summary statistics for each population are provided in Table S3. The tests for HWE assuming an alternative hypothesis of heterozygote deficiency yielded only four significant outcomes among 115 tests. Conversely, the other alternative hypothesis of heterozygote excess provided no significant outcomes. micro-cheker suggested the presence of null alleles at these three loci: *Sfo*12, *Sfo*177 and *Sfo*D105. Three populations significantly deviated from Hardy–Weinberg equilibrium: namely MET, JC and LDE (Table S3). All pairwise *F*_ST_ estimates across all loci were significant after a Bonferroni correction (*P* < 0.05; Table S4). This translated into a global *F*_ST_ value of 0.120 ± 0.064, reflecting a moderate overall level of differentiation among populations.

### Bayesian clustering analyses

**Figure 1:**
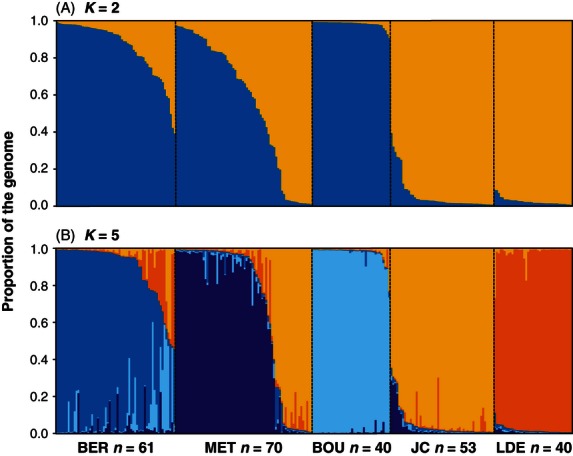
Individual genomic proportion assigned by structure to each population sample based on 23 microsatellites genotypes. (A) *K* = 2 corresponds to the uppermost level of structuring between the domestic (light orange) versus wild genomic background (light blue). (B) *K* = 5 corresponds to the differentiation between the five populations: Petit Saint-Bernard Lake (BER; light blue), Méthot Lake (MET; dark blue), Bourassa Lake (BOU; turquoise), Jacques Cartier aquaculture facility (JC; light orange) and Lac des Écorces aquaculture facility (LDE; dark orange). The *y*-axis depicts the genomic proportion belonging (co-ancestry) to one of the populations from either the stocked populations (BER or MET) or the reference populations (BOU, JC or LDE). Each column corresponds to an individual, and sample locations are separated by vertical dotted bars.

Using structure for the comparison between the reference and stocked populations, the Δ*K* statistic (Evanno et al. 2005) showed the highest peaks for *K* = 2 and a smaller one for *K* = 5 (Table S5). The two clusters with the most pronounced structure differences corresponded to the wild (BER, BOU, MET) versus domestic (JC, LDE) populations used for stocking (Fig. 1A). The five clusters inferred corresponded to the five distinct populations: BER, BOU, JC, LDE and MET (Fig. 1B). structure clustering also confirmed that both BER and MET populations included fish of variable levels of admixture between wild and domestic genetic backgrounds, whereas strains used for stocking were essentially homogeneous (Fig. 1). Overall, individual admixture proportions in each stocked population were confidently estimated with narrow 90% credibility interval surrounding their mean posterior probability (Fig. S1). For *K* = 2, the admixture between wild and domestic backgrounds in the two stocked lakes were 0.143 ± 0.148 for BER, and 0.442 ± 0.352 for MET (Table 1). Admixture values for *K* = 5 were similar as for *K* = 2, the mean admixture from the LDE domestic background in BER was 0.108 ± 0.153 and that of the JC domestic background in MET was 0.311 ± 0.403 (Table 1). Mean admixture from BOU was estimated at 0.090 ± 0.151 in BER, and 0.020 ± 0.038 in MET (Table 1). The weak admixture value in MET may indicate the presence of background noise in the genetic signal, since BOU individuals have not been stocked in that lake.

**Table 1:** Results summary of the structure program for *K* = 2 and *K* = 5. The following thresholds were used to assess hybrid status: individuals were considered F1 or post-F1 hybrids when: 0.20 ≤ *Q* ≤ 0.80; as wild individuals when: 0.00 ≤ *Q* < 0.20 and domestic when: 0.80 < *Q* ≤ 1.00. Mean admixture (±standard deviation) was calculated for *K* = 5 as the addition of individual admixture proportions from the two domestic backgrounds (JC and LDE). The range of *Q* values is given below the mean domestic admixture

	*K* = 2	*K* = 5
BER (*n* = 61)		
Mean domestic	0.143 ± 0.148 (0.008–0.609)	0.108 ± 0.153 (0.004–0.543)
Mean LDE		0.071 ± 0.125
Mean JC		0.038 ± 0.092
Mean BOU		0.090 ± 0.151
MET (*n* = 70)		
Mean domestic	0.442 ± 0.352 (0.026–0.991)	0.311 ± 0.403 (0.005–0.994)
Mean LDE		0.037 ± 0.065
Mean JC		0.274 ± 0.391
Mean BOU		0.020 ± 0.038

BER, Petit Saint-Bernard Lake; MET, Méthot Lake; BOU, Bourassa Lake; JC, Jacques Cartier aquaculture facility; LDE, Lac des Écorces aquaculture facility.

### Fulton's condition index

The nonlinear regression equation obtained from all fish combined was *W*_*i*_ = 9.0 × 10^−06^ × *L*_*i*_^3.03^ with *R*^2^ = 0.96 (Fig. S2; linear regression after log_10_ transformation of the mass and the fork length: *F* = 3043.40, *P* < 0.001). This confirmed the isometric relationship between the weight and length (*b* ≍ 3). The mean condition index was 1.06 ± 0.16 in BER and 1.17 ± 0.19 in MET. The mean condition index for fish with a high domestic genetic background (0.80 < *Q* ≤ 1.00) was 1.18 ± 0.21, whereas it was 1.07 ± 0.16 for fish with a high wild genetic background (0.00 ≤ *Q* < 0.20). Thus, the GLS model revealed a positive relationship between the Fulton's condition index and levels of domestic introgression (*F* = 58.68, *P* < 0.001). Also, fish sampled in 2008 had a higher condition than those collected in 2009 (*F* = 375.48, *P* < 0.001). There was also an interaction between year and lake, due to individuals in MET having a higher condition index than BER in 2009 (*F* = 5.06, *P* = 0.026).

### Patterns of gene expression

There were no significant second-order interactions among variables and therefore all interactions terms were removed from subsequent analyses. The level of introgression, defined as the proportion of domestic genetic background estimated with *K* = 2, had an overall significant effect on the gene expression (*F* = 2.79, *P* = 0.011), as it was also the case for *K* = 5 (*F* = 2.68, *P* = 0.015). For simplicity of interpretation, only the model with domestic admixture estimated with *K* = 2 was considered in subsequent analyses, as it represented the most pronounced structure differences corresponding to the wild versus domestic background when using the Δ*K* statistic. Moreover, mean admixture values for *K* = 2 and *K* = 5 were slightly different but comparable; we assume *K* = 2 as representative. Sex effect was only marginally non-significant (*F* = 1.80, *P* = 0.098) and was removed from the final model, whereas year of sampling, lake, sampling method and Fulton's condition index all had significant effect in the overall model.

**Figure 2:**
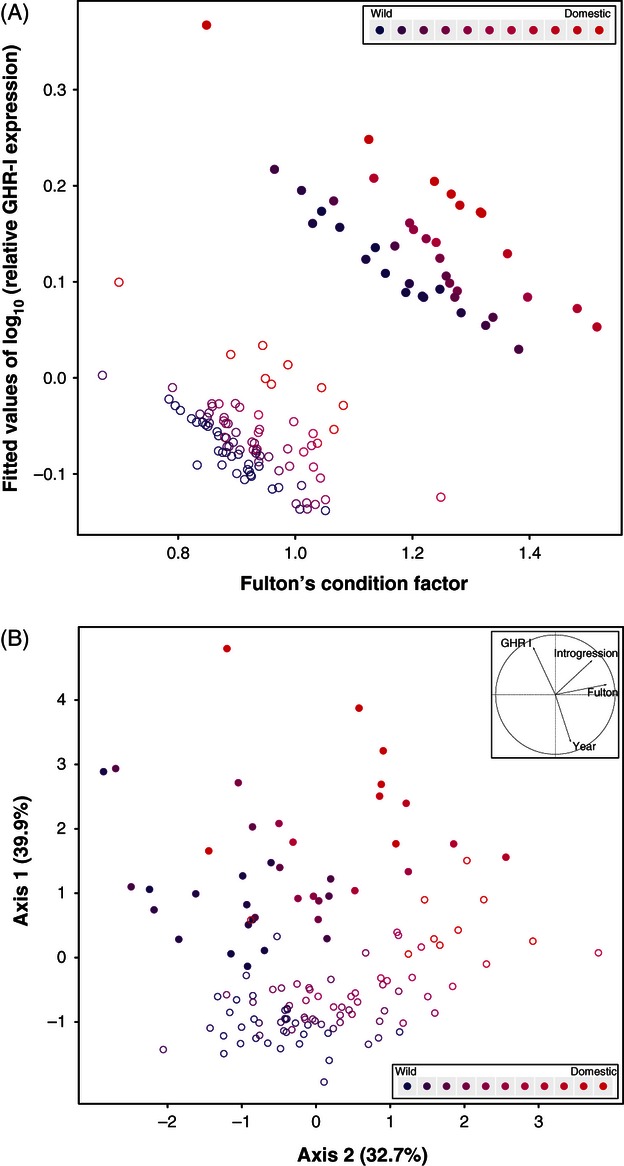
Illustration of the growth hormone receptor isoform I (GHR-I) expression, taking into account the three significant independent variables from the manova model (introgression, Fulton's condition index and year of sampling). (A) Fitted values of the GHR-I expression from the manova model as function of Fulton' condition index. GHR-I expression and Fulton's condition index can be depicted on the *y*- and *x*-axis respectively, whereas year of sampling is involved in difference of the intercepts; (B) a factorial analysis of mixed data representation of the GHR-I expression. Filled and unfilled circles correspond to the sampling year 2008 and 2009, respectively. Colours from blue to red represent the continuous admixture proportion for each individual from wild to domestic (0.00 ≤ *Q* ≤ 1.00).

The level of domestic introgression had a significant net effect (independent of other factors) on expression of two genes: GHR-I (*P* = 0.048) and COX VIIa (*P* = 0.041; Table 2). GHR-I expression was systematically higher in 2008 than 2009 and increased with the level of introgression (*F* = 3.98; *P* = 0.048; *y* = *a* + 0.125*x*; SE = 0.062; Fig. 2A). There was also a significant negative relationship between GHR-I and Fulton's condition index when taking year into account (*F* = 5.91; *P* = 0.016; *y* = *a* + −0.383*x*; SE = 0.158). The FAMD results for the GHR-I are illustrated in the Fig. 2B. The GHR-I expression, year and introgression were the main factors loading on the first FAMD axis (Table 3), which explained 39.9% of the variance. In agreement with the manova model (Fig. 2A), the FAMD showed that the level of GHR-I expression was generally higher for fish sampled in 2008 than 2009 and secondly, it revealed an increase in GHR-I expression as a function of domestic introgression for each year. The Fulton's condition index and introgression loaded on the second FAMD axis (Table 3) and explained 32.7% of the variance, revealing a positive correlation between the two factors, in accordance with the GLS model.

**Table 2:** Results for each gene obtained from the manova model with *K* = 2 from structure. Intercepts are given for categorical independent variables (lake, year and sampling method) and slope of linear relationships for continuous independent variables (introgression and Fulton's condition index), only when significant. Reference intercepts for lakes = Méthot Lake, for years = 2009, sampling methods = contrast of gillnets/angling versus trap nets

		Estimates			
				
Coefficients	Genes	Slopes	Intercepts	*F* values	*P* values
Introgression	Apolipoprotein			0.70	0.404
	**Cytochrome c oxidase VIIa**	0.096		4.28	0.040
	**Growth hormone receptor I**	0.125		3.98	0.048
	Heat shock protein 90			0.03	0.853
	Insulin growth factor I			0.33	0.568
	Metallothionein			2.60	0.109
	Transferrin			0.09	0.768
Lake	Apolipoprotein			3.43	0.066
	Cytochrome c oxidase VIIa			1.52	0.220
	Growth hormone receptor I			0.24	0.623
	**Heat shock protein 90**		−0.121	17.38	<0.001
	**Insulin growth factor I**		0.246	16.97	<0.001
	**Metallothionein**		−0.173	11.24	0.001
	Transferrin			2.11	0.149
Year of sampling	**Apolipoprotein**		−0.240	4.73	0.031
	**Cytochrome c oxidase VIIa**		−0.106	5.36	0.022
	**Growth hormone receptor I**		−0.301	23.62	<0.001
	**Heat shock protein 90**		−0.294	49.03	<0.001
	Insulin growth factor I			0.12	0.734
	**Metallothionein**		−0.288	14.94	<0.001
	Transferrin			0.01	0.951
Fulton's condition index	Apolipoprotein			0.26	0.615
	Cytochrome c oxidase VIIa			0.06	0.807
	**Growth hormone receptor I**	−0.384		5.91	0.016
	**Heat shock protein 90**	−0.253		5.59	0.020
	**Insulin growth factor I**	−0.479		4.73	0.032
	Metallothionein			0.17	0.686
	Transferrin			0.08	0.785
Sampling method	Apolipoprotein			1.23	0.297
	**Cytochrome c oxidase VIIa**		−0.102	3.64	0.029
	Growth hormone receptor I			0.30	0.743
	Heat shock protein 90			1.36	0.260
	**Insulin growth factor I**		−0.309	12.73	<0.001
	Metallothionein			1.97	0.143
	Transferrin			0.65	0.526

Significance (*P* values) for the fixed effects was obtained through a type III test (*F* statistic). Significant genes are in bold characters.

**Table 3:** Percentage of variance explained by each variable on each axis for the two FAMD analyses

Analysis	Variables	Axis 1	Axis 2
I	Growth hormone receptor I	38.87	10.39
	Introgression	20.36	28.69
	Fulton's condition Index	1.78	55.86
	Year of sampling	38.99	5.06
II	Cytochrome c oxidase VIIa	28.85	2.42
	Introgression	4.05	42.94
	Sampling method	29.58	54.64
	Year of sampling	37.52	≈0.00

**Figure 3:**
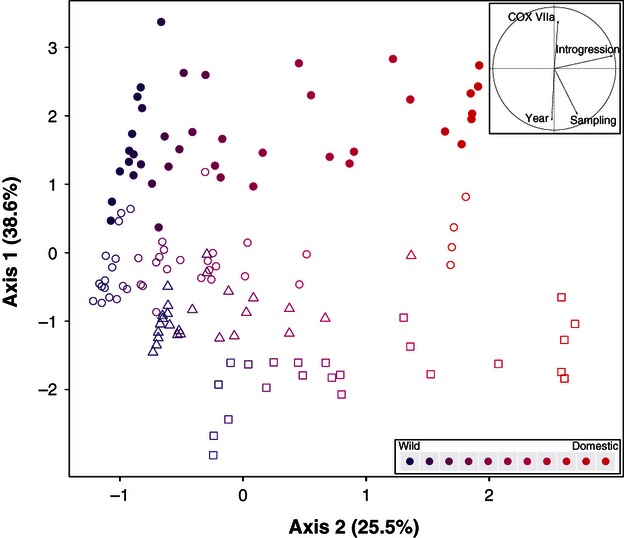
A factorial analysis of mixed data (FAMD) representation of the cytochrome c oxidase VIIa (COX VIIa) expression and its interaction with the three significant independent variables from the manova model (introgression, year of sampling and sampling methods). Filled and unfilled symbols represent the years of sampling 2008 and 2009, respectively. Colours from blue to red represent the continuous admixture proportion for each individual from wild to domestic (0.00 ≤ *Q* ≤ 1.00). Symbols represent the sampling method used: circle, triangle and square stand for gillnets, angling and trap nets, respectively. Fish were only caught by gillnets in 2008 but all three methods were used in 2009.

The FAMD results for the COX VIIa expression as a function of the three significant independent variables from the manova model (introgression, year of sampling and method of sampling) is presented in Fig. 3. Level of COX VIIa expression, year of sampling and sampling method all loaded on the first FAMD axis (Table 3), which explained 38.6% of the variance. As for GHR-I, COX VIIa expression was higher for fish sampled in 2008 than 2009. In 2009, the FAMD also revealed an overall higher level of COX VIIa expression for fish caught by gillnets relative to those caught by trap nets Indeed, trap net was significantly different from the two other sampling techniques (*t* = 2.58, *P* = 0.011; *t* = 2.58, *P* = 0.024), but no difference was observed between gillnet and angling (*t* = 0.08, *P* = 0.94) following contrast tests. Thirdly, within a given year for each sampling method, the FAMD also showed that the level of COX VIIa expression was positively influenced by domestic introgression, independent of the year and method of sampling (*F* = 4.28, *P* = 0.040; *y* = *a* + 0.096*x*; SE = 0.046). The introgression factor loaded on FAMD axis two (Table 3), which explained 25.5% of the variance, suggesting that the level of introgression was not strongly influenced by the year of sampling. Sampling method also loaded on this axis (Table 3).

The level of domestic introgression had no effect on the expression of genes other than GHR-I and COX VIIa (Table 2). However, other genes were influenced by other variables included in the model (Table 2). Namely year of sampling had a significant effect on the expression of all genes except transferrin and insulin growth factor I whereby all genes were more expressed in 2008 than 2009 (Table 2). The lake effect was also significant whereby heat shock protein 90 and metallothionein showed higher level of expression in BER, whereas insulin growth factor I showed a higher level of expression in MET (Table 2). Fulton's condition index had a significant effect on the expression of heat shock protein 90 and insulin growth factor I, with the level of expression of these two genes being negatively correlated with Fulton's condition index (Table 2). Finally, sampling had a significant effect on the expression of insulin growth factor I (Table 2), with a significantly lower expression for fish that were caught with the trap nets when compared to the two other sampling techniques (*t* = 4.14, *P* < 0.001; *t* = 4.90, *P* < 0.001; for the gillnet and angling comparison, respectively). However, no significant differences were observed between the gillnets and angling (*t* = 1.71, *P* = 0.090) following contrasts tests.

## Discussion

The main objective of this study was to assess the impacts of domestic introgression on the expression profile of seven candidate genes in two stocked brook charr populations in natural conditions. Our analyses revealed that the level of expression of two of the genes tested, COX VIIa and the GHR-I, was significantly positively correlated with the level of introgression. While the expression of the other five genes was not significantly affected by introgression, it was nevertheless influenced by other variables, except for transferrin. Namely, we observed an overall higher level of transcription for most genes in 2008 versus 2009. Our results also showed that the sampling method affected gene expression, whereby the level of expression tended to increase with the suspected level of stress associated with the method of capture, from fish trap nets to angling and gillnets. There was also a lake effect on three of the genes. Finally, we observed a positive association between the level of introgression and Fulton's condition index suggesting that in natural conditions, domestic introgression may impact on the physiological conditions of introgressed wild fish. To our knowledge, this is the first demonstration in nature that introgression from stocked populations have an impact on the expression of genes playing a role in important biological functions that may be related with fitness.

The observation of a positive correlation between the level of domestic introgression and the level of expression of the GHR-I gene in brook charr corroborates the recent observation in rainbow trout of an increase in the level of expression of GHR with the proportion of domestic alleles (Tymchuk et al. 2009). This result is also in agreement with previous microarray studies, which reported the overexpression of GHR in domesticated relative to wild strains in several salmonid species (Devlin et al. 2009; Normandeau et al. 2009; Bougas et al. 2010). For instance, farmed Atlantic salmon in Canada showed a 23% increase of expression of the GHR gene compared to their respective wild counterpart (Roberge et al. 2006). Furthermore, growth hormone transgenesis and manipulation in salmonids provided evidence of concordant GHR upregulation along with artificial selection, when compared with wild counterpart or untreated fish (Gahr et al. 2008; Devlin et al. 2009). Tymchuk et al. (2009) also suggested a pivotal role of the GHR in the growth hormone/insulin growth factor I axis in fish liver. In addition, growth activation in Atlantic salmon coincided with increased GH receptor gene expression (Wargelius et al. 2005). All of these studies thus support the crucial role of this gene in the regulation of growth and show that artificial selection for growth has led to rapid genetic change in GHR expression among domestic strains versus wild populations of several salmonid species. Previous studies in Atlantic salmon and brook charr showed that only three to seven generations of domestication led to significant changes in transcription profiles of GHR as well as many other genes involved in various biological functions (Roberge et al. 2006; Sauvage et al. 2010). As the domestic population used in Québec has undergone at least 15 generations of domestication (Bougas et al. 2010), it is plausible that some of the differentiation has been generated by directional selection for traits of commercial interest (e.g. growth). Here, we could not compare growth among fish with different levels of domestic admixture because the fish were not aged. However, Fulton's condition index was also correlated with the extent of domestic introgression, suggesting a genetic impact on phenotypic variation of a fitness-related trait in wild fish. Admittedly, however, whether or not this influences positively or negatively the fitness of wild brook charr remains to be investigated.

This study also revealed a positive correlation between domestic introgression and the expression of the COX VIIa. COX VIIa gene codes for a subunit of the cytochrome c oxidase protein belonging to the complex IV in the oxidative phosphorylation pathway in the mitochondria, which is thus implicated in the energy metabolism. In contrast to our results, cytochrome c oxidase family genes were generally underexpressed in farmed relative to wild fry Atlantic salmon and rainbow trout (Roberge et al. 2006; Tymchuk et al. 2009). A subsequent microarray analysis in brook charr also reported under expression of COX VIIa when comparing two wild populations with the same domestic strain we analysed here (JC) (Bougas et al. 2010). Indeed, Sauvage et al. (2010) found that cytochrome b complex subunit 1, a gene involved in the upstream electron chain of the complex III, was also downregulated after three generations of selection. Overall, a reduction in expression of genes involved in energy production could be consistent with a reduction of the basal metabolic rate of farmed fish favouring allocation of resources towards growth, or due to oxidative damage to mitochondria (Roberge et al. 2006; Eya et al. 2012). Given these observations, an increase of COX VIIa expression with the level of domestic introgression in wild populations appears counterintuitive. We see at least four possible nonexclusive hypothetical explanations for such result. First, non-parallelism is often observed in the expression of complex and polygenic traits such as gene involve in oxidative phosphorylation pathway. For instance, previous studies performed on another salmonid, the lake whitefish (*Coregonus clupeaformis*), found evidence for gene transcription upregulation of cytochrome c genes (complex IV) in the dwarf relative to the normal population in Cliff Lake (Maine), whereas an opposite pattern was observed in other lakes (Derome et al. 2006; Evans and Bernatchez 2012). Secondly, most of the previous studies were based on the analysis of whole body juvenile fish (Tymchuk et al. 2009), which may have buffered the expression level of this cytochrome c oxidase subunit from the liver, given that the level expression of a given gene may vary substantially among tissues (Eya et al. 2012). Also, patterns of expression are expected to vary between life-history stages (Nolte et al. 2009). Finally, fish with high domestic genetic background may perhaps compensate the lower food supply in natural condition by actively increasing their metabolism rate.

Besides highlighting the effect of introgression on levels of expression of GHR-I and COX VIIa genes, the results of this study also revealed that other variables, namely year of sampling and lake of origin, had a significant impact on the expression of all the candidate genes, except for the transferrin gene. All genes showed a lower level of gene expression in 2009 than 2008. An obvious environmental parameter that could explain inter-annual differences is water temperature. Although temperature data for the whole growing season were not available for both lakes sampled, surface temperature was recorded at the time of sampling. In both lakes, mean surface temperature at time of sampling was much higher in 2008 (BER: 20°C and MET: 16.5°C) than in 2009 (BER: 14°C and MET: 8°C). Increased HSP 90 gene expression, coding for a heat shock protein, is of particular interest since heat shock proteins are known to be good indicators of cellular stress responses due to their chaperone capacitor activity during the highly conserved heat shock response (Feder and Hofmann 1999). Recently, HSP 90 has been shown to be a good candidate gene for thermal tolerance in arctic charr (*Salvelinus alpinus*) for which an upregulation was observed during thermal stress, with thermo-tolerant arctic charr overexpressing HSP 90 when compared to intolerant fish (Quinn et al. 2011). Similarly, during the reproductive season of sockeye salmon (*Oncorhynchus nerka*), members of the 90-, 70- and 40-kDa families of heat shock proteins were all upregulated in the gill and liver tissues in response to temperature stress in the riverine environment (Evans et al. 2011). Temperature could also have affected the GHR-I gene, as shown in previous studies under laboratory conditions. For instance, juvenile rainbow trout fed *ad libitum* in high water temperature (16°C) showed higher levels of both GHR isoforms in liver than fish kept at low water temperature (8°C; Gabillard et al. 2006b). Reduction of food availability has been shown to cause downregulation in GHR-I and GHR-II genes in gilthead sea bream (*Sparus aurata*; Saera-Vila et al. 2005) and zebra fish (*Danio rerio*) liver tissues (Drew et al. 2008). Interestingly, COX VIIa was also downregulated with starvation in this latter study. Although growth data were not available in our study, we observed a trend for fish collected in 2008 to have a higher condition index relative to fish collected in 2009. This may reflect better growing conditions associated with higher temperature in 2008.

Differences in gene expression between BER and MET lakes were observed for HSP 90, metallothionein and IGF-I. The IGF-I gene expression pattern contradicted the temperature hypothesis, as it was upregulated in the colder MET Lake. Differences between lakes could reflect adaptive differentiation given their relatively high level of genetic divergence (*F*_ST_ = 0.111). Since IGF-I can be considered as a good indicator of growth (reviewed in Beckman 2011), overexpression of IGF-I in the MET population could suggest that individuals in the MET population grow faster than in the BER population. However, this cannot be assessed further in the context of this study. Nevertheless, it is noteworthy that genetically differentiated Atlantic salmon populations exhibited variation in their global gene expression (Tymchuk et al. 2010). In addition, in *Fundulus heteroclitus*, variation in expression for 13 genes was found to be under selection for habitat temperature than could be accounted by genetic distance alone (Whitehead and Crawford 2006).

We also documented a positive association between the level of domestic introgression and Fulton's condition index. Interestingly, this tendency was also observed in a previous experiment, where the same domestic strain had always a higher body mass compared to other wild and hybrid strains in different rearing environments (Crespel et al. 2012). Although more accurate measurements of fitness are warranted, our results hint at a potential fitness advantage for introgressed fish should improved physiological status reflected by a higher condition index translate into improved survival and/or reproductive success. Nevertheless sampling period or environmental variations could also modulate the association between domestic introgression, Fulton's condition index and GHR-I expression. In addition, a negative relationship was observed between the expressions of two genes, IGF-I and HSP 90, and the condition index. One explanation for these intriguing results is that gene upregulation in wild genetic background could be associated with compensatory growth and overcome constitutive gene expression, which is associated with a normal growth rate. For example, after a period of starvation, Atlantic salmon and rainbow trout switched to a fast growth rate and overexpressed GHR-I in liver tissue and IGF-I in muscle tissue when compared to control fish (Chauvigné et al. 2003; Gabillard et al. 2006a; Bower et al. 2008). During growth stimulated by IGF-I, chaperonin capacitor activity for proper folding of newly synthesized proteins may be required. Indeed, during recovery growth, Atlantic salmon and rainbow trout overexpressed HSP genes in muscle, indicating activation of unfolded protein response pathways (Rescan et al. 2007; Bower and Johnston 2010). Thus, association between the expression of IGF-I, GHR-I and HSP 90 and the condition index may reveal complex trade-offs between energetic reserves and/or growth and feeding status of brook charr in natural conditions.

We also observed an overexpression of COX VIIa and IGF-I in fish caught by angling and gillnetting, compared to fish caught by trap nets. In rainbow trout, distinct stressors (high or low temperature, re-used water, salinity and handling/confinement) resulted in different level of plasma cortisol, with the highest values obtained for low temperature and re-used water (Sánchez et al. 2011). While we did not measure the plasma cortisol here, the expression level of these two genes tended to increase with the suspected increase of stress associated with the sampling techniques, like gillnetting and angling when compared to fish trapping. A previous microarray study in rainbow trout is in accordance with an upregulation of gene involved in electron transport, insulin-like growth factor receptor pathway and insulin-like growth factor within an hour and up to 24 h post-exposure to a 3 min handling disturbance (Wiseman et al. 2007). Also, in juvenile gilthead sea bream, IGF-I was significantly down-expressed in liver only after 24 h post-confinement stress (Saera-Vila et al. 2009). These studies thus suggest that the magnitudes of differences of both COX VIIa and IGF-I gene expression between sampling techniques may reflect transcriptional characteristic of the early stress response.

The multivariate model revealed no significant sex-linked effects on the expression of candidate genes tested. This result was somewhat unexpected since previous research, using expression QTL (eQTL) mapping and qPCR experiments, has reported a sex-biased transcriptional genetic architecture in brook charr (Côté et al. 2007; Sauvage et al. 2012a). We suggest that the marginally non-significant sex effect in our model could be due to the presence of multiple cohorts with different developmental stages. Also, 27 immature individuals could not be sexed at the time of sampling, which reduced our power to detect a possible sex effect in our analyses.

Finally, we did not detect any introgression effect on five of seven candidate genes. This observation can be multifactorial, from the candidate gene selection up to the statistical analysis. First, candidate genes were selected on the basis of comparisons of different wild populations and domestic strains than those investigated here, as well as from Atlantic salmon (Roberge et al. 2008; Normandeau et al. 2009; Bougas et al. 2010; Sauvage et al. 2010). It is thus possible that observed differences between wild and domestic fish in these previous studies were not replicable here. For instance, hybridization, from a genomic perspective, is a stochastic and independent event leading to different epistatic interactions and genetic modes of allelic interactions (e.g. dominance, transgressivity, etc.) likely to result in un-predictable gene expression impacts of a different qualitative and quantitative order depending of the wild population introgressed with domestic alleles (Normandeau et al. 2009; Bougas et al. 2010). Also, these previous studies were performed in controlled laboratory environment that may not be representative of natural conditions, and thus translated into differential patterns of expression in controlled versus natural environments. In addition, the analyses of these previous studies were based on the analysis of early life stages in contrast to the use of either older juvenile or adult fish in this study. Differential patterns of gene expression between different life stages, which have previously been documented in other salmonids (Nolte et al. 2009), could thus have resulted in contrasted results among studies. Admittedly also, our analysis may have suffered from statistical power limitation due to either the limited sample size that was available, or to the reduced signal-to-noise ratio due to additional sources of environmental variation.

To conclude, this study confirmed that introgression can alter patterns of gene transcription and that this was repeatable in two consecutive years despite different thermal conditions. Our data also suggested that the introgression impacts could be effective from the first and second generation of hybrids as previously documented (Bougas et al. 2010). These results expand our understanding of interdependent gene expression interacting with environmental factors, together controlling complex processes linked to the growth and physiological status. Future studies should aim at assessing the (i) epigenomic interactions during introgressive hybridization, which may modulate the expression of phenotypic traits (i.e. gene expression), and (ii) relationships between gene expression, fitness (in terms of survival and reproductive success) and population dynamics to provide a clearer assessment of the eco-evolutionary dynamics in partially admixed populations (Carroll 2011).

From a management perspective, this study showed that the combination of gene expression and admixture analyses greatly contributes to our understanding of the functional impacts of introgressive hybridization following the introduction of domestic individuals in wild populations. These results re-emphasize the need to restrict stocking activities using domestic strains to already extensively introgressed populations regarding the potential consequences on the physiology of locally adapted populations (Marie et al. 2010). Also, those lakes should not be connected and be in the lower portion of drainage to restrain potential migration. We recommend that pristine populations should not be stocked to maintain potential local adaptation by applying rigorous management plan and policies. Those would not necessarily be incompatible with the sport fishery industry since such practices would increase the market (in terms of offering a wild ‘pristine product’) and conservation values of those populations. Also, the physiological consequences of gene expression patterns are not yet fully elucidated and raise fundamental conservation questions, such as: can we consider introgressed populations as lost ‘pure’ natural populations, with little relevance for conservation (Carroll 2011)?

## Data archiving statement

Data for this study are available at Dryad: doi:10.5061/dryad.qf488.

## Supporting Information

**Figure S1.** Individual admixture proportion with the 90% confidence interval based on 23 microsatellites using the structure software.

**Figure S2.** Relationship between the mass (g) and the fork length (mm) of all the fish sampled in Petit Saint-Bernard Lake and Méthot Lake for both years 2008 and 2009.

**Table S1.** Multiplex PCR protocole.

**Table S2.** Sequencing primers, real-time PCR primers, and Taqman minor groove binder probe for each candidate gene.

**Table S3.** Descriptive genetic analyses for each population, the number of fish genotyped (N), allelic richness based on minimum sample size of 37 individuals (*Ar*), observed (*H*_*O*_) and expected (*H*_*E*_) heterozygosity and *F*_IS_.

**Table S4.** Pairwise genetic differentiation (*F*_ST_) based on 23 microsatellite markers.

**Table S5.** structure statistic output and the ad hoc statistic of Evanno et al. (2005) to determine the best *K* for all comparisons.
