# Supplementary material for: Stocking impacts the expression of candidate genes and physiological condition in introgressed brook charr (Salvelinus fontinalis) populations
Source: Evol Appl. 2012 Oct 23;6(2):393–407. doi: 10.1111/eva.12022 (PMC3586627; doi:10.1111/eva.12022)
Supplement: Supplementary file 1 [file eva0006-0393-SD1.doc]

**Supplementary materials**

**
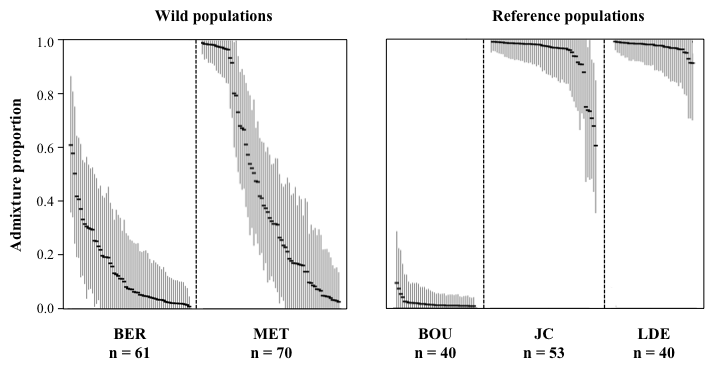
**

**Figure S1.** Individual admixture proportion with the 90% confidence interval based on 23 microsatellites using the Structure software. Results are presented for *K* = 2, corresponding to the uppermost level of structuring between the domestic vs. wild genomic background. The y-axis depicts the proportion of the genome belonging to domestic background. The reference populations correspond to two stocking strains (Jacques Cartier Hatchery (JC), Lac des Ecorces Hatchery (LDE)) and one wild reference population (Bourassa Lake (BOU)). The two wild populations are Methot Lake (MET) and the Petit St. Bernard Lake (BER).


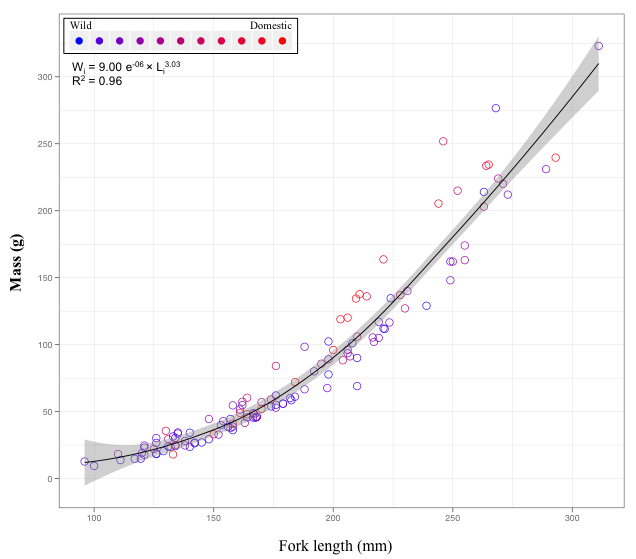


**Figure S2.** Relationship between the mass (g) and the fork length (mm) of all the fish sampled in BER and MET for both years 2008 and 2009. Colour represents the continuous admixture proportion for each individual from wild to domestic (0.00 ≤ Q ≤ 1.00). The line ± 95% confidence interval was derived from the nonlinear regression W_i_ = aL_i_^b^.

**Table S1.** Multiplex PCR protocole

| Multiplex pairs | Microsatellite | Final concentration of primers in 10 μl  (pmol) | Annealing temperature |
| --- | --- | --- | --- |
| 1 | *Sfo*B52 | 5.6 | 64°C |
|  | *Sfo*C28 | 4.2 |  |
|  | *Sfo*C88 | 1.4 |  |
|  | *Sfo*C113 | 2.8 |  |
|  |  |  |  |
| 2 | *Sfo*266 | 16.8 | 60°C |
|  | *Sfo*C24 | 0.7 |  |
|  | *Sfo*C86 | 0.7 |  |
|  | *Sfo*D75 | 1.4 |  |
|  | *Sfo*D100 | 0.7 |  |
|  |  |  |  |
| 3 | *Sco*216 | 2.8 | 64°C |
|  | *Sco*218 | 5.6 |  |
|  | *Sfo*262 | 5.6 |  |
|  |  |  |  |
| 4 | *Sfo*269 | 7 | 60°C |
|  | *Sfo*308 | 4.2 |  |
|  | *Sfo*D91 | 9.8 |  |
|  | *Sfo*D105 | 2.8 |  |
|  |  |  |  |
| 5 | *One*8 | 0.84 | 60°C |
|  | *Sfo*12 | 2.52 |  |
|  | *Sfo*177 | 6.3 |  |
|  | *Sfo*226 | 2.8 |  |
|  | *Sfo*C115 | 2.1 |  |
|  | *Ssa*85 | 0.7 |  |
|  | *Ssa*197 | 0.7 |  |

**Table S2.** Sequencing Primers, Real-Time PCR Primers, and Taqman MGB Probe for Each Candidate Gene. Product sizes are given for the brook charr (*Salvelinus fontinalis*) partial cDNA sequences generated with the sequencing primers.

| Genes | Sequencing primers (5’ – 3’)  Forward  Reverse | Product size (bp) | Real-Time PCR Primers (5’–3’)  Forward  Reverse | Taqman MGB Probe  (5’–3’) | Product size  (bP) |
| --- | --- | --- | --- | --- | --- |
| Apolipoprotein | GGCTGCCATGCTAACCTGTTCTAT  TTCTGCTTCAGCTCGTCAGTGT | 524 | CACCGTCCAGATGATCAGGAA  CTGGCCACATCAGCACTCTCT | AGTTGGGACAGGATGTC | 78 |
| β Actin | AGATGAAATCGCCGCACTGGTT  CTCGTTGTAGAAGGTGTGATGCCA | 278 | GCTGTCTTCCCCTCCATCGT  TCTCCCACGTAGCTGTCTTTCTG | TCGTCCCAGGCATC | 83 |
| Cytochrome c oxidase VIIa | AGCAGCAAGAGGCTTCACAC ACATGCATTAGCAGGGCTTC | 301 | AAGGGAGGGACCACTGATGTC  TGCCTGCGAGGGTGATG | TTCTCTACCGTCTAACAAT | 80 |
| Elongation factor Iα | TGGGTGAGTTTGAGGCTGGTATCT  TGACGGACACGTTCTTGACGTT | 594 | GCCCCTCCAGGATGTCTACA  ACGGCCCACGGGTACTG | ATCGGCGGTATTGGA | 55 |
| Heat shock  protein 90 | ATGACTGGGAGGAACACCTG CCAGCTTGAGGTTCTTGGAG | 390 | GTCTTCATCATGGACAGCTGTGA  CCACACCACGCACAAAGTTC | CTCATCCCAGAGTACC | 61 |
| Metallothionein | TGCTCTAAAACTGGCTCTTGCAAC  CTAGGCTCAAGATGGTACAACCAA | 235 | CGCATGCACCAGTTGTAAGAA  CACACAGCCTGAAGCACATTTACT | AGTTGCTGCCCCTGC | 76 |
| Transferrin | GAGCTCCCATCGACAGCTAC CTGGCATTCGATCCTTGATT | 395 | GACGTTGCCTTCATCAAGCA  GCAACTCATAGCTTGCCTTCTCT | CTGGCTGTACCTGCC | 61 |
| Insulin growth factor I |  |  | CAGGCATCCAGATTGTGCAA  ACCATGTTCTGAGAATTCCTGTGTT | CAGCCATTACTCTCTG | 75 |
| Growth hormone receptor I |  |  | CCCACTGCCCCCTGTATCT  CTTCAGAAGGAGGCTGTTTTGC | ACCATGGTGGAAGGAG | 69 |

**Table S3.** Descriptive genetic analyses for each population, the number of fish genotyped (N), allelic richness based on minimum sample size of 37 individuals (*Ar*), observed (*H_O_*) and expected (*H_E_*) heterozygosity and *F*_IS_. *Hardy-Weinberg disequilibrium after correction for multiple testing.

| Groups | Populations | N | *Ar* | *H_E_* | *H_O_* | *F*_IS_ |
| --- | --- | --- | --- | --- | --- | --- |
| Portneuf | Méthot (MET) | 70 | 10.93 | 0.766 | 0.722* | 0.065 |
|  |  |  |  |  |  |  |
| Mastigouche | Petit St Bernard (BER) | 61 | 9.730 | 0.717 | 0.704 | 0.027 |
|  |  |  |  |  |  |  |
| References | Bourassa (BOU) | 40 | 6.895 | 0.520 | 0.528 | -0.002 |
|  | Jacques Cartier (JC) | 53 | 10.64 | 0.748 | 0.729* | 0.034 |
|  | Lac Des Écorces (LDE) | 40 | 6.801 | 0.677 | 0.666* | 0.030 |

**Table S4.** Pairwise genetic differentiation (*F*_ST_) based on 23 microsatellite markers. All comparisons are significant (at *P*<0.05) after 1000 permutations over loci.

|  |  | Portneuf | | Mastigouche | | |
| --- | --- | --- | --- | --- | --- | --- |
|  |  | JC | MET | BER | BOU | LDE |
| Portneuf | JC |  |  |  |  |  |
|  | MET | 0.047 |  |  |  |  |
| Mastigouche | BER | 0.068 | 0.111 |  |  |  |
|  | BOU | 0.152 | 0.239 | 0.165 |  |  |
|  | LDE | 0.077 | 0.080 | 0.136 | 0.239 |  |

**Table S5.** Structure statistic output and the ad hoc statistic of Evanno et al. (2005) to determine the best *K* for all comparisons. (*K*) The number of populations assumed in the Structure analysis. Mean LnP(*K*): The mean posterior probability of the data over 10 runs for each *K*. Stdev LnP(*K*): The standard deviation of the posterior probability of the data for a given *K*. Ln'(*K*): The mean difference between *K* and *K*-1 likelihood values. |Ln''(*K*)| the absolute value of the difference between Ln'(*K*+1) and Ln'(*K*) values. Delta *K* The mean of the absolute values of |Ln''(*K*)| averaged over 10 runs divided by the standard deviation of LnP(*K*). Boldness indicates the *K* selected based on Delta *K*.

| K | Mean LnP(K) | Stdev LnP(K) | Ln'(K) | |Ln''(K)| | Delta K |
| --- | --- | --- | --- | --- | --- |
| 1 | -24130.57 | 1.54 | NA | NA | NA |
| **2** | **-22463.75** | **4.46** | **1666.82** | **829.07** | **185.75** |
| 3 | -21626.00 | 96.32 | 837.75 | 155.96 | 1.62 |
| 4 | -20944.21 | 50.56 | 681.79 | 44.93 | 0.89 |
| **5** | **-20307.35** | **18.18** | **636.86** | **618.30** | **34.01** |
| 6 | -20288.79 | 144.71 | 18.56 | 93.37 | 0.65 |
| 7 | -20363.60 | 504.89 | -74.81 | 107.21 | 0.21 |
| 8 | -20331.20 | 338.21 | 32.40 | 876.58 | 2.59 |
